# The Effect of Job Skill Demands Under Artificial Intelligence Embeddedness on Employees’ Job Performance: A Moderated Double-Edged Sword Model

**DOI:** 10.3390/bs14100974

**Published:** 2024-10-21

**Authors:** Ningning Chen, Xinan Zhao, Lele Wang

**Affiliations:** 1 School of Business Administration, Northeastern University, Shenyang 110167, China; 1810446@stu.neu.edu.cn (N.C.); xnzhao@mail.neu.edu.cn (X.Z.); 2 Party School of Liaoning Provincial Party Committee, Shenyang 110004, China

**Keywords:** artificial intelligence, job skill demands, competency needs, job embeddedness, well-being in organizations, job performance, technological anxiety

## Abstract

With the widespread application of AI technology, the skills and abilities required by employees in their work are undergoing fundamental changes, redefining the roles of employees. This research aims to explore the effect of job skill demands under AI embeddedness on well-being in organizations and job performance. Based on conservation of resources theory, this research randomly selected 479 employees from 8 companies in China using a time-lag method as samples, and conducted statistical analysis with ordinary least squares (OLS). This research found that, job skill demands under AI embeddedness will both increase employees’ competency needs, promoting their well-being in organizations and job performance and decrease employees’ job embeddedness, inhibiting their well-being in organizations and job performance. Meanwhile, technological anxiety moderated the impact of job skill demands under AI embeddedness on job embeddedness.

## 1. Introduction

With the rapid development of technology, artificial intelligence (AI) has deeply penetrated into organizational management work with its unique processing and learning capabilities, profoundly affecting organizational development and employee work [1,2,3]. Artificial intelligence technology has become an important skill for survival in the workplace [4,5]. As an innovative driving force, AI can be combined with big data and various industry sectors, not only improving production efficiency, driving social, economic, and technological development, but also posing new demands for employees’ work methods, profoundly impacting the nature and content of their work [6,7].

Firstly, AI technology can help employees handle repetitive and mechanical tasks, improve employees’ work efficiency [8], and enhance product quality and output. Secondly, the development of AI technology has given birth to many emerging industries, such as driverless cars, smart homes, and personalized healthcare [9,10,11]. These new industries not only promote technological progress but also create a large number of employment opportunities and promote the rapid development of economy and society.

Previously, organizations focused on employees’ skills in traditional professional knowledge, communication abilities, and leadership skills. However, with the widespread application of AI technology, the skills and abilities required by employees in their work are undergoing fundamental changes [12,13]. Firstly, employees need to have stronger technological adaptability, especially the ability to adapt to and operate AI tools, automation systems, data analysis platforms [14]. Secondly, the ability to cooperate with AI technology. With the popularity of automation and AI, employees’ work will shift more towards interaction and cooperation with AI technology, such as interaction with robots, automated processes, and intelligent assistants [15]. Thirdly, the ability of continuous learning. As AI technology continues to evolve, many emerging skills and jobs will change within months or years. Employees cannot rely on traditional “single skills” and need to continuously learn and improve their various skills and knowledge [16].

AI technology was widely used in production manufacturing, management decisions, customer service, and organizational strategic planning. Firstly, in production manufacturing, AI technology improves production efficiency and precision through automating production processes [17]; Secondly, in management decision-making, AI technology helps management identify trends and patterns by analyzing historical data, thus making more accurate decisions [18]; Thirdly, in customer service, AI technology can analyze customers’ feedback and comments to help enterprises optimize service quality and product design [8]; Fourthly, in organizational strategic planning, AI technology can analyze market trends, competitors and customer needs to provide data support for enterprises’ long-term strategies [19].

With the popularization and application of AI, traditional skill models are being challenged, requiring employees to have higher levels of innovative thinking and human-machine collaboration skills, redefining the roles of employees [20,21]. This is not only to cope with increasingly complex tasks but also to maintain a competitive advantage in a human-machine collaborative work environment. Therefore, how to enhance employees’ well-being in organizations and job performance, is not only an inevitable requirement for organizations to adapt to technological changes but also a key factor for employees to achieve personal career development [22].

Therefore, the application of AI technology in organizational management has become a focus of attention in both academia and practice. Currently, research has delved into the impact of AI technology on employee skill demands and job performance [23,24], but there is little literature discussing the mechanism of how employee skill demands under the perspective of AI technology embeddedness affect employees’ well-being in organizations and job performance, that is, how employee job skill demands under the perspective of AI technology embeddedness affect employees’ well-being in organizations and job performance through what pathways, and what factors affect the degree of their relationship. Understanding this mechanism is crucial for employees to adjust their behavior under the impact of AI technology, improve their well-being in organizations and job performance, and promote sustainable development of enterprises.

The Conservation of Resources Theory (COR) provides a theoretical framework for exploring the impact mechanism of job skill demands on employees’ well-being in organizations and job performance under AI embeddedness from the perspectives of resource gain and resource consumption [25]. Skill demands under the perspective of artificial intelligence embeddedness change employees’ work methods and content, requiring employees to have higher levels of innovation thinking and human-machine collaboration skills, which have a significant impact on employees’ individual resources [26].

On one hand, skill demands under the perspective of artificial intelligence embeddedness require employees to continuously learn and adapt to new technologies and tools [27], which can stimulate employees’ competency needs, enable employees to acquire more psychological resources, and when competency needs are met, employees may experience higher job satisfaction, thereby increasing their individual resources and promoting the sustainable development of employee job performance.

On the other hand, job skill demands under the perspective of artificial intelligence embeddedness are seen as a resource burden [28], as workers must consumes a large amount of time and energy to acquire the necessary skills and adjust to emerging technologies and tools, which undoubtedly consumes a large amount of individual resources, reduces employees’ well-being in organizations, thereby lowering the sustainable performance level of employees.

The COR theory also points out that individuals’ cognitive evaluation and emotional responses to resources are also influenced by their own characteristics, resulting in varying degrees of cognition and reactions [29]. Therefore, the impact of job skills demands from the perspective of AI embeddedness on employees’ well-being in organizations and job performance largely depends on the extent of employees’ technological anxiety towards artificial intelligence.

Technological anxiety refers to individuals’ anxiety and concerns about technology use and coping with technological changes [30]. Employees who has higher levels of technological anxiety are more likely to resist and reject advanced technologies like artificial intelligence, which can decrease their willingness to accept and learn about new technologies compared to those with lower levels of technological anxiety [31], significantly lowering their level of job embeddedness and competency needs, thereby negatively impacting employees’ well-being in organizations and job performance. Therefore, we believe that technological anxiety moderates the mechanism through which job skills demands affect individuals’ well-being in organizations and job performance under the perspective of artificial intelligence embeddedness.

Therefore, this study delves into the mechanism of how job skill demands affect employees’ well-being in organizations and job performance under AI embeddedness, not only having important theoretical implications for understanding the “black box” of job skill demands on employees’ well-being in organizations and job performance under AI embeddedness, but also having important practical significance for how to formulate effective strategies to enhance employees’ adaptability to AI technology and maximize its role in the workplace.

This study collected data through questionnaire survey, participants were all asked to use AI assistants to complete their jobs. To enhance the causality between variables, this study collected data in three stages, and one month between each survey. The first survey (T1) included employees’ demographic characteristics, independent variables (job skills demands), and moderating variables (technological anxiety); the second survey (T2) included mediating variables (competency needs, job embeddedness, and well-being in organizations); the third survey (T3) included the dependent variable (job performance).

This study bridges the one-sidedness of previous researches that have only explored the positive or negative effect of job skill demands from a single perspective of AI-embedded perspectives, revealing the double-edged sword effect of job skill demands on well-being in organizations and job performance under AI embeddedness, and responding to the inconsistency of previous research results. Meanwhile, this study can help managers within organizations fully understand the benefits and drawbacks of AI usage. The AI usage may have positive or negative effects on employees’ psychology, behavior, and collaborative job performance. Therefore, organizational managers need to arrange the application of AI reasonably according to different types of organizations, different employees, and different job contents, to maximize the benefits of AI and prevent any adverse consequences.

This study mainly includes four parts. The first part was hypothesis development. The second part determines the research methods, specifically including participants and procedure as well as measurement tools. The third part is empirical analysis, specifically including common method bias test, descriptive statistical analysis, confirmatory factor analysis and hypothesis testing. The fourth part is discussion, specifically including theoretical implications, practical implications, limitations and future research directions.

## 2. Hypothesis Development

### 2.1. Positive Pathway: The Mediating Role of Competency Needs

#### 2.1.1. Job Skills Demands and Competency Needs under AI Embeddedness

COR suggests that employees must actively acquire, utilize, preserve different resources within their work environments to effectively manage job demands and attain high performance. Job skill demands, as an external demands, has a large effect on the resources that employees need in their work [32], thus affecting the extent to which their competency needs are met. On the one hand, regarding the integration of artificial intelligence, job skills demands necessitate employees to continually learn and adapt to emerging technologies and tools, enabling them to acquire more new knowledge and skills that can assist them in better completing their work, thereby increasing their competency needs [33]. On the other hand, employees acquiring more new knowledge and skills will increase their psychological resources. According to COR, individuals will invest resources to acquire more resources, thus further stimulating their competency needs. Hypothesize the following:

**H1.**  *Job skill demands under AI embeddedness is positively associated with competency needs*.

#### 2.1.2. Competency Needs and Well-Being in Organizations

Competency needs can be seen as a resource, and individuals will try to satisfy these demands to the fullest extent possible, thereby increasing their sense of well-being [34]. On the one hand, the satisfaction of competence demands can increase an individual’s confidence in their work. The COR theory suggests that individuals tend to maximize the use of existing resources to minimize resource loss. When individuals feel competent in the tasks and responsibilities required for their work, they will face work challenges with more confidence, making it easier for them to feel satisfied and happy [35]. Therefore, satisfying competence demands can enhance employee well-being; on the other hand, the satisfaction of competence demands helps individuals achieve their own values and development goals [36]. The COR theory suggests that individuals usually choose behaviors that can satisfy their demands to the greatest extent possible to achieve personal goals and values. Therefore, in the work environment, satisfying competence demands means that individuals can fully utilize their abilities and skills, achieve work goals, and receive recognition. This sense of achievement can significantly increase employee well-being in organizations. In summary, hypothesize the following:

**H2.**  *Competency needs is positively associated with well-being in organizations*.

#### 2.1.3. Well-Being in Organizations and Job Performance

On the one hand, based on COR theory, individuals with abundant resources will make resource investments to acquire more resources. Therefore, employees with higher well-being have more psychological resources [37]. In order to obtain more individual resources, employees will invest more of their individual resources into their work, thereby improving their job performance. On the other hand, when employees have higher well-being, they are more inclined to cooperate with colleagues, share their knowledge and resources [38], and thus form a mutual supportive and motivating work environment, thereby increasing their job performance [39]. In conclusion, hypothesize the following:

**H3.**  *Well-being in organization is positively associated with job performance*.

#### 2.1.4. The Chain Mediating Role of Competency Needs and Well-Being in Organizations

According to COR theory, job skills demand is positively associated with competency needs under AI embeddedness. When employees’ competency needs are met, they will experience more well-being in organizations, which means that employees possess more psychological resources. At this point, based on the COR theory, individuals will make resource investments in order to acquire more resources. Therefore, employees with higher well-being in organizations will invest more individual resources into their work, effectively utilizing and leveraging their skills, thereby enhancing their job performance. Therefore, we believe that the job skill demands have an indirect positive impact on employees’ job performance by increasing their competency needs and well-being in organizations under the AI embeddedness. In conclusion, hypothesize the following:

**H4.**  *Competency needs and well-being in organizations play positively mediating role in the impact process of job skill demands on job performance under the AI embeddedness*.

### 2.2. Negative Pathway: The Mediating Role of Job Embeddedness

#### 2.2.1. Job Skill Demands and Job Embeddedness

Job embeddedness directs individuals to avoid leaving their work due to a combination of factors [40,41]. COR theory proposes that individuals accumulate and expend resources within their work environments, and these resources play a crucial role in job embeddedness. In a work environment embedded with artificial intelligence, work may become more complex and diverse, requiring employees to continuously learn new knowledge and technologies related to artificial intelligence, and to constantly adjust to adapt to new ways of working [42]. This process may consume a large amount of employees’ resources, including energy, time, and cognitive resources [43]. According to COR theory, when individuals face resource loss, they will activate their own resource defense mechanisms to reduce their own resource loss. Therefore, faced with the job skill demands from the perspective of artificial intelligence embeddedness, individuals may feel resource scarcity, reduce their resource investment in work, and consequently lower their level of job embeddedness. In conclusion, hypothesize the following:

**H5.**  *Job skill demands under AI embeddedness is negatively associated with job embeddedness*.

#### 2.2.2. Job Embeddedness and Well-Being in Organizations

Firstly, when individuals feel closely connected to their job tasks, organization, and work environment, it can enhance their commitment and sense of belonging to the job [44]. This makes them more willing to invest time and effort to complete tasks, experience more meaning and satisfaction from their work, and ultimately increase their well-being in organizations [45]. Secondly, job embeddedness provides employees with more social opportunities and support, enhancing their sense of belonging and identification with colleagues and superiors, leading to a positive work state and emotional experience [46], which in turn contributes to further increasing their well-being in organizations. Finally, the task richness and challenge in job embeddedness enhance employees’ motivation and autonomy, allowing them to showcase their abilities and talents at work, satisfy their autonomy demands, and increase their well-being in organizations [47]. In conclusion, hypothesize the following:

**H6.**  *Job embeddedness is positively associated with well-being in organizations*.

#### 2.2.3. The Chain Mediating Effect of Job Embeddedness and Well-Being in Organizations

It is clear from the preceding reasoning that job skill demands may is negatively associated with job embeddedness under the AI embeddedness. A lower level of job embeddedness will significantly reduce employees’ well-being in organizations, and well-being in organizations is an important factor affecting employees’ job performance [48].Therefore, combining the above assumptions, we believe that job embeddedness and well-being in organizations play a chain-mediated role in the impact process of job skill demands on employees’ job performance under AI embeddedness. In conclusion, hypothesize the following:

**H7.**  *Job embeddedness and well-being in organizations play negatively mediated role in the impact process of job skill demands on employees’ job performance under AI embeddedness*.

### 2.3. The Moderating Effect of Technological Anxiety

Based on the COR theory, individuals accumulate and expend resources within their work contexts, and these resources are pivotal for achieving well-being within organizations and sustaining job performance over time [29]. Technological anxiety may affect individuals’ acquisition and depletion of resources in meeting job skill demands and job embeddedness processes [30], thereby impacting the influence of job skill demands on individuals’ well-being in organizations and job performance under the context of AI embeddedness. On the one hand, technological anxiety may affect individuals’ acquisition of resources in meeting job skill demands and job embeddedness processes. In the context of artificial intelligence embeddedness, changes in job skill demands may result in individuals needing to acquire new knowledge and skills to meet job demands. However, technological anxiety may cause individuals to develop fear and anxiety towards technological change, hindering their active acquisition of new resources [49]. Technological anxiety may lead individuals to develop resistance and aversion towards new technologies and job demands [50], making it difficult for them to acquire relevant resources in meeting job demands and job embeddedness processes, thereby reducing their competence and well-being in organizations.

On the other hand, technological anxiety may result in individuals depleting their resources in meeting job skill demands and job embeddedness processes [51]. In a work environment with artificial intelligence embeddedness, changes in job skill demands may require employees to continuously learn new knowledge and technologies, adapt to new ways of working, which will undoubtedly consume a large amount of individual resources. Individuals with higher levels of technological anxiety may feel greater work pressure and resource depletion, and in order to prevent further loss of their own resources, employees may initiate their own resource defense mechanisms, reducing their competence and level of job embeddedness.

In conclusion, technological anxiety may moderate the impact of job skill demands on competency needs and job embeddedness under the AI embeddedness. Therefore, hypothesize the following:

**H8.**  *Technological anxiety negatively moderates the impact of job skill demands under the AI embeddedness on the competency needs*.

**H9.**  *Technological anxiety positively moderates the impact of job skills demands on job embeddedness under the AI embeddedness*.

**Figure 1:**
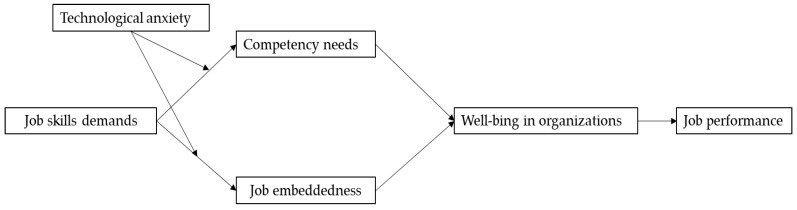
Research model. Source: Prepared by this study.

In summary, this research develops a model (refer to Figure 1) that examines how job skill demands affect individuals’ well-being in organizations and job performance from the perspective of AI embeddedness.

## 3. Methods

### 3.1. Research Process

**Figure 2:**
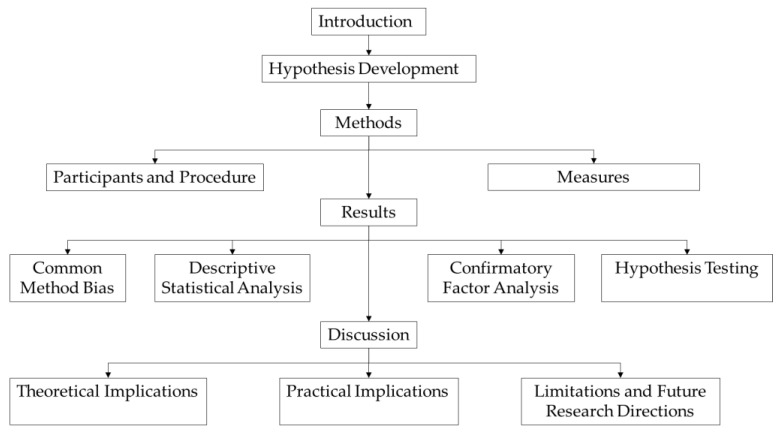
Process of this study. Source: Prepared by this study.

This study follows the following study procedures (Figure 2). Firstly, under the in troduction, we propose the hypotheses of this study based on the Conservation of Resources Theory (COR) and related researches. Secondly, determine the research methods, this part mainly includes participants, procedure and measures. Subsequently, we conduct empirical tests on the obtained data, mainly including common method bias analysis, descriptive statistical analysis, confirmatory factor analysis and hypothesis testing. Finally, we discussed the research results, mainly including theoretical implications, practical implications, research limitations and future research directions.

### 3.2. Pre-Test

To assess the quality of the questionnaire, we conducted a pre-test on employees of two hotel enterprises in Liaoning Province. Eventually, 78 valid questionnaires were recovered.

We conducted reliability analysis and validity analysis. The results of the independent *t*-test show that all designed questions have reached the significance level required for differentiation. In addition, the Cronbach’s alphas of job skills demand under AI embeddedness, competency needs, job embeddedness, well-being in organizations, job performance, and technological anxiety were 0.902, 0.889, 0.864, 0.912, 0.879, and 0.907. This indicates that the data has good reliability. To obtain the validity of the study, Kaiser-Meyers-Olkin (KMO) was used. It was found that the validity of this questionnaire is within an acceptable range, indicating a high structural validity [52]. The results show that the questionnaire data passed all these tests and was suitable for formal research.

### 3.3. Participants and Procedure

Through two weeks of continuous communication and exchange, a total of 8 Chinese companies have committed to providing assistance for the field research of this study. Among the 8 companies, 3 are hotel companies, 3 are manufacturing companies, and 2 are education companies. For the convenience of data matching, researchers assigned a unique number to each employee participating in the survey: for example, the first employee from initial company is given the number 1001, the next employee is given 1002; the first employee from the second company is given the number 2001, the second employee is given 2002, and the pattern continues. Additionally, aim to increase employee participation, researchers prepared exquisite small gifts and distributed them to the participating employees before distributing the questionnaires. The first survey was conducted on 16 December 2023, with 800 questionnaires distributed and 708 collected; The second survey was conducted on 16 January 2024, with 682 questionnaires distributed and 613 collected; The third survey was conducted on 26 February 2024 with 576 questionnaires distributed and 531 collected. Finally, 479 usable surveys were collected, the overall response rate was 59.875%. Many statisticians suggest that the sample size should be 5 to 10 times the number of items in the questionnaire [53], so the sample size in this study was valid.

In the accurate data set, 56.785% of the employees were male, and 43.215% were female. Among the employees, those with a bachelor’s degree made up the largest group at 37.552%. The average age of the participants was 31.7 years, and the average tenure was 6.2 years.

### 3.4. Measures

The survey in this study was designed using the Likert 5-point scale.

(1) Job skill demands under AI embeddedness. We utilize the scale created by Zhu et al. [54] and Karasek [55] to evaluate the level of job skill demands, including items such as “My job skills have improved with the introduction of AI technology or equipment”. The Cronbach’s α was 0.880.
(2) Competency needs. We utilize the scale created by Ilardi et al. [56] to evaluate the level of competency needs, involves such as “I am able to handle my job”. The Cronbach’s α was 0.909.
(3) Job embeddedness. This study utilize the measurement scale created by Crossley et al. [57] to evaluate the level of job embeddedness, including items such as “It would be difficult for me to leave this job”. The Cronbach’s α was 0.960.
(4) Well-being in organizations. We utilize the scale created by Zheng et al. [38] to evaluate the level of well-being in organizations, involves such as “Overall, I am very satisfied with the work I do” The Cronbach’s α was 0.948.
(5) Job performance. We utilize the measurement scale created by Borman and Motowidlo [58] to evaluate the level of job performance, including items such as “I am able to fulfill my responsibilities at work sustainably”. The Cronbach’s α was 0.885.
(6) Technological anxiety. We utilize the scale created by Cidral et al. [59] to evaluate the level of technological anxiety, including items such as “Service robots make me feel uneasy and confused”. The Cronbach’s α was 0.944.
(7) Control variables. Some scholars find that gender, age, education, and tenure can impact employees’ well-being in organizations [37,38] and job performance [60,61]. Therefore, this study will treat the above variables as control variables.

## 4. Results

### 4.1. Common Method Bias

We used Harman’s Single-Factor Test to test the extent of common method bias, the results showed that the maximum factor variance explained of 26.323%, below the 50% standard. This suggests that there is no significant issue of this study.

### 4.2. Descriptive Statistical Analysis

Table 1 displays the results of descriptive statistical analysis. Firstly, Job skill demands under AI embeddedness is positively associated with competency needs (r = 0.257, *p* < 0.01); Competency needs is positively associated with well-being in organizations (r = 0.135, *p* < 0.01); Well-being in organization is positively associated with job performance (r = 0.637, *p* < 0.01). The positive pathway proposed in this study was basically supported, laying a foundation for in-depth exploration of the mediating role of competency needs and well-being in organizations. Secondly, Job skill demands under AI embeddedness is negatively associated with job embeddedness (r = −0.164, *p* < 0.01), Job embeddedness is positively associated with well-being in organizations (r = 0.126, *p* < 0.01). The negative pathway proposed in this study was basically supported, laying a foundation for in-depth exploration of the mediating role of job embeddedness and well-being in organizations. Thirdly, we found that age and education have a significant impact on employees’ well-being in organizations. Meanwhile, tenure has a significant impact on employees’ job embeddedness and technology anxiety.

In empirical research, the mean is widely used to describe the central tendency of data and is an important indicator for describing the overall characteristics of data. In this study, job skill demands under AI embeddedness, competency needs, and job performance are all at a higher level, while job embeddedness and well-being in organizations are at a moderate level, and technological anxiety is at a lower level. The standard deviation is an indicator that reflects the degree of dispersion of data and represents the average distance between data points and the mean. In this study, the standard deviation is between 0.749 and 1.096, indicating that the data in this study are relatively concentrated but still have variability.

### 4.3. Confirmatory Factor Analysis

This study utilized Mplus7.4 software to perform a confirmatory factor analysis on job skill demands, competency needs, job embeddedness, technological anxiety, well-being in organizations, and job performance to examine the discriminant validity between variables. The results (see Table 2), Model 1 had the best fit, and significantly outperformed the other 5 models, suggests that there is strong evidence of distinctiveness among the six primary variables examined in this research.

### 4.4. Hypothesis Testing

Initially, we examined the positive pathway. According to Figure 2, there is a notable beneficial effect of job skill demands on competency needs (β = 0.317, SE = 0.055, *p* < 0.001), H1 received confirmed; competency needs have a notable beneficial effect on well-being in organizations (β = 0.182, SE = 0.051, *p* < 0.001), H2 received confirmed; well-being in organizations have a notable beneficial effect on job performance (β = 0.420, SE = 0.017, *p* < 0.001), H3 received confirmed. Then, we test the chain mediation effect of Competency needs and well-being in organizations. Job skill demands have a significant indirect positive impact on job performance through competency needs and well-being in organizations (β = 0.024, SE = 0.010, 95% [0.006, 0.044]), thus H4 was supported. By testing H1, H2, H3, and H4, the positive path proposed in this study was supported.

**Figure 3:**
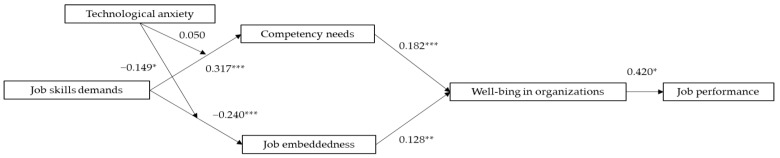
Path coefficients of the model. Source: Prepared by this study. Notes: * *p* < 0.05, ** *p* < 0.01, *** *p* < 0.001.

Afterward, we examined the negative pathway. According to Figure 3, the job skill demands have a notable adverse effect on job embeddedness (β = −0.240, SE = 0.065, *p* < 0.001), H5 received confirmed; Job embeddedness has a notable beneficial effect on well-being in organizations (β = 0.128, SE = 0.044, *p* < 0.01), H6 received confirmed. At the same time, we test the chain mediation effect of job embeddedness and well-being in organizations. Job skill demands have a notable indirect adverse effect on job performance through job embeddedness and well-being in organizations (β = −0.013, SE = 0.006, 95% CI [−0.027, −0.003]), H7 received confirmed. By testing H3, H5, H6, and H7, the negative path proposed in this study was supported.

In addition, in empirical research, the coefficient size usually reflects the degree of influence of the independent variable on the dependent variable. The larger the coefficient, the stronger the influence of the independent variable on the dependent variable. In this study, the positive influence of job skill demands under AI embeddedness on competency needs is stronger than the negative influence of job skill demands under AI embeddedness on job embeddedness. At the same time, the positive influence of competency needs on well-being in organizations is stronger than the positive influence of job embeddedness on well-being in organizations.

**Figure 4:**
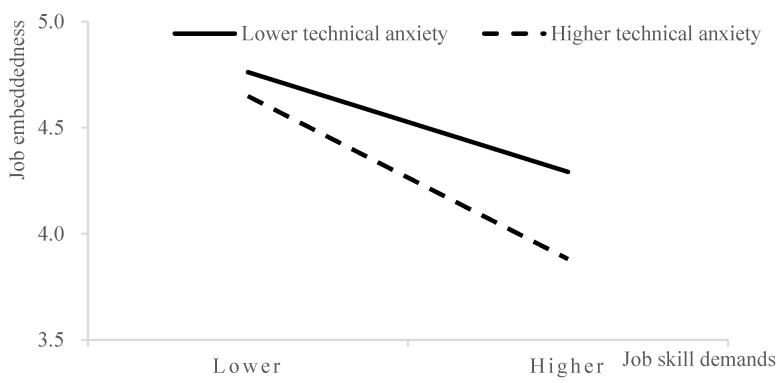
The Moderating effect of technological anxiety. Source: Prepared by this study.

Finally, we explored the moderating effect of technological anxiety (see Table 3). According to Model 2, the interaction between job skill demands and technological anxiety has no notable impact on competency needs (β = −0.050, *p* > 0.05), H8 did not receive confirmed. According to Model 4, the interaction between job skill demands and technological anxiety has a notable effect on job embeddedness (β = −0.149, *p* < 0.05), H9 received confirmed. In addition, in order to more clearly demonstrate the moderating effect of technological anxiety, we conducted a simple slope analysis. As shown in Figure 4, when technological anxiety at higher level, the negative connection between the job skills demands and the level of job embeddedness will be more pronounced, while when technological anxiety at lower level, the relationship will be weaker. Therefore, H9 was supported again. In addition, we found that tenure has a significant negative impact on job embeddedness in the empirical model. This indicates that tenure has an important influence on employees’ job embeddedness. Therefore, tenure is an important control variable when exploring the influence mechanism of job embeddedness, the introduction of tenure can improve the explanatory power and accuracy of the influence mechanism of job skill demands under AI embeddedness on job embeddedness.

## 5. Discussion

### 5.1. Theoretical Implications

Firstly, we explored the double-edged sword effect of job skill demands on employees’ well-being in organizations and job performance under the AI embeddedness. Currently, researchers have individually studied the benefits and drawbacks of job skill demands from various angles, leading to conflicting or inconclusive findings [27,62], while ignoring the potential double-edged sword effect of job skill demands under the AI embeddedness [63,64]. This study bridges the one-sidedness of previous researches that have only explored the positive or negative effect of job skill demands from a single perspective of AI embeddedness, revealing the double-edged sword effect of job skill demands on well-being in organizations and job performance under AI embeddedness, and responding to the inconsistency of previous research results.

Secondly, we explored the boundary conditions of the double-edged sword effect of job skill demands on well-being in organizations and job performance under the perspective of artificial intelligence embeddedness. The COR theory points out that individuals’ cognitive evaluations and emotional responses to resources are also influenced by their own characteristics, thus showing varying degrees of cognition and response [29,65]. Therefore, the impact of job skill demands on well-being in organizations and job performance under the perspective of artificial intelligence embeddedness may largely depend on the degree of employees’ technological anxiety towards artificial intelligence. We found that technological anxiety moderated the impact of job skill demands on job embeddedness. That is, faced with job skill demands under the AI embeddedness, employees with higher technological anxiety have lower job embeddedness compared to those with lower technological anxiety. However, the moderating effect of technological anxiety between job skills demands and competency needs is not significant. Our research results validate and enrich previous academic studies on technological anxiety [30,66,67].

Finally, this study further confirms the COR theory. With the popularization of AI, many scholars have explored the impact of artificial intelligence use on individual behavior and job performance from the perspectives of resource gain and resource loss, and have achieved fruitful results [22,68]. Building on previous research, we once again verified the dual-edged sword effect of job skill demands on well-being in organizations and job performance under AI embeddedness from the perspectives of resource gain and loss, and further confirmed the moderating effect of technological anxiety on the dual-edged sword effect. Therefore, this study further validates the COR theory.

### 5.2. Practical Implications

Firstly, managers within organizations must fully understand the benefits and drawbacks of AI usage. The AI usage may have positive or negative effects on employees’ psychology, behavior, and collaborative job performance. Therefore, organizational managers need to arrange the application of AI reasonably according to different types of organizations, different employees, and different job contents, to maximize the benefits of AI and prevent any adverse consequences.

Secondly, organizational managers should pay attention to the impact of job skill demands on well-being in organizations and job performance under the perspective of artificial intelligence embeddedness. On the one hand, job skill demands will increase employees’ competency needs, thereby increasing their well-being in organizations and job performance under the perspective of artificial intelligence embeddedness. On the other hand, job skill demands will decrease employees’ job embeddedness, thereby reducing their well-being in organizations and job performance under the perspective of artificial intelligence embeddedness. Therefore, when organizations introduce artificial intelligence assistants to assist employees in their work, organizational managers can enhance employees’ competency needs and improve their human-machine collaboration capabilities by providing relevant training and necessary work support, thereby avoiding a decrease in the level of job embeddedness and ultimately enhancing employees’ well-being in organizations and job performance.

Finally, managers need to fully recognize the significant impact of employees’ own technological anxiety. We found that technological anxiety moderates the influence of job skill demands on individual job embeddedness under the AI embeddedness. Therefore, organizational managers must focus on the impact of technological anxiety on employees’ psychological, behavioral, and job performance in their daily management work. On the one hand, relevant technical and knowledge training can be imposed to reduce employees’ technological anxiety; on the other hand, during the recruitment process, the technological anxiety of employees can be assessed, and employees with strong adaptability to new knowledge and new technologies and strong learning abilities can be recruited.

### 5.3. Limitations and Future Research Directions

Initially, Job skill demands will affect employees’ psychology, behavior, and job performance through a series of psychological mechanisms under AI embeddedness. We focused on how competency needs and job embeddedness influence employees’ well-being in organizations and job performance. Future studies could investigate additional factors that play a role in how job skill demands impact employees’ well-being and job performance within the context of AI integration, further enriching the relevant research content.

Secondly, although this research employed a research design with multiple time points and confirmed through various statistical methods that there is no significant common method bias present in this study, self-report questionnaires may still have common method bias issues. Future research can involve organizational managers in evaluating employees’ job performance or use other methods such as the experimental method to enhancing the reliability of research conclusions.

Finally, even if the study shows a statistically significant effect, if the effect size is small, the practical significance may be limited, rendering the findings less useful in real-world applications [69]. In this study, some coefficients are less than 0.3, which may lead to relatively low practical validity. The reasons for this result may mainly be the following two: Firstly, the sample size is small, resulting in estimation bias of coefficients [70]; secondly, important independent variables related to the dependent variable may be omitted, which may lead to a low regression coefficient [71]. In future research, should balance practical validity and statistical validity as much as possible to enhance the practical impact of the research.

## 6. Conclusions

With the popularization and application of artificial intelligence, traditional skill models are being challenged, requiring employees to have higher levels of innovative thinking and human-machine collaboration skills, and repositioning employees [72,73,74]. This study constructs a dual-edge model of the impact of job skill demands on well-being in organizations and job performance under the AI embeddedness. We found that job skill demands under the AI embeddedness have a dual-edge impact on well-being in organizations and job performance by increasing competency needs and reducing job embeddedness.

Meanwhile, technological anxiety moderates the relationship between job skill demands on job embeddedness. In other words, individual who has higher level of technological anxiety is less likely to feel job embeddedness when faced with job skill demands related to artificial intelligence, compared to employees with lower levels of technological anxiety. However, the moderating effect of technological anxiety between job skill demands and competency needs is not significant. The reason for this result may be that employees with competency needs do not experience a significant amount of technological anxiety when facing job skill demands under the perspective of artificial intelligence embeddedness [75].

## Figures and Tables

**Table 1:** Dscriptive statistical analysis.

Variables	1	2	3	4	5	6	7	8	9	10
1. Gender	1									
2. Age	−0.004	1								
3. Education	−0.086	−0.053	1							
4. Tenure	0.084	0.002	−0.040	1						
5. Job skill demands	−0.020	0.012	−0.117 *	0.038	1					
6. Competency needs	0.022	0.060	−0.062	−0.025	0.257 **	1				
7. Job embeddedness	0.069	−0.001	−0.043	−0.120 **	−0.164 **	−0.054	1			
8. Well-being in organizations	0.001	−0.104 *	0.133 **	−0.041	−0.028	0.135 **	0.126 **	1		
9. Job performance	−0.009	−0.006	0.073	−0.026	0.213 **	0.648 **	−0.140 **	0.637 **	1	
10. Technological anxiety	0.030	−0.042	0.033	−0.108 *	−0.090 *	−0.204 **	0.142 **	0.075	−0.064	1
Mean	1.390	2.875	2.770	3.480	3.697	3.624	3.083	3.289	3.552	2.271
SD	0.487	0.873	0.758	1.159	0.761	0.943	1.096	1.044	0.749	0.967

Notes: * *p* < 0.05, ** *p* < 0.01. Source: Prepared by this study.

**Table 2:** Confirmatory factor analysis.

Modle	χ^2^/df	RMSEA	GFI	CFI	TLI	IFI
1. Six Factors (including 1, 2, 3, 4, 5, 6)	2.596	0.056	0.901	0.960	0.951	0.961
2. Five Factors (including 1 + 2, 3, 4, 5, 6)	4.012	0.089	0.840	0.848	0.821	0.849
3. Fore Factors (including 1 + 2 + 3, 4, 5, 6)	11.106	0.143	0.642	0.733	0.705	0.734
4. Three Factors (including 1 + 2 + 3 + 4, 5, 6)	15.893	0.179	0.543	0.592	0.551	0.592
5. Tow Factors (including 1 + 2 + 3 + 4 + 5, 6)	19.824	0.199	0.488	0.499	0.454	0.501
6. Single Factors (including 1 + 2 + 3 + 4 + 5 + 6)	24.936	0.224	0.424	0.361	0.306	0.362

Source: Prepared by this study.

**Table 3:** Testing of moderation effects.

Variables	Competency Needs		Job Embeddedness	
	Model 1	Model 2	Model 3	Model 4
Gender	0.081	0.086	0.095	0.099
Age	0.069	0.076	0.003	0.010
Education	−0.057	−0.011	−0.069	−0.063
Tenure	−0.019	−0.019	−0.127 **	−0.107 **
Job skill demands		0.331 ***		−0.235 ***
Technological anxiety		−0.116 **		−0.262 ***
Job skill demands * Technological anxiety		0.050		−0.149 *

Notes: * *p* < 0.05, ** *p* < 0.01, *** *p* < 0.001. Source: Prepared by this study.

## Data Availability

The data supporting the findings of this study can be obtained by contacting the first author.
